# Durability of Exercise vs. Revascularization in Intermittent Claudication: An Updated Meta-Analysis of Randomized Trials Focusing on Patient-Centered Outcomes

**DOI:** 10.3390/healthcare14020170

**Published:** 2026-01-08

**Authors:** Mislav Puljevic, Petra Grubic-Rotkvic, Mia Dubravcic-Dosen, Andrija Stajduhar, Majda Vrkic-Kirhmajer

**Affiliations:** 1 School of Medicine, University of Zagreb, 10000 Zagreb, Croatia; 2 University Hospital Centre Zagreb, 10000 Zagreb, Croatia; 3 Andrija Štampar School of Public Health, University of Zagreb, 10000 Zagreb, Croatia

**Keywords:** PAD, exercise, claudication

## Abstract

Intermittent claudication (IC) is the most frequent symptomatic manifestation of lower-extremity peripheral artery disease (PAD). Supervised exercise therapy (SET) and endovascular revascularization (ER) are established treatments, but their relative and combined effects on health-related quality of life (HRQoL) remain. We conducted a systematic review and meta-analysis of randomized controlled trials (RCTs) comparing SET, ER, and ER+SET, with HRQoL as the primary outcome. **Methods**: Following PRISMA 2020, PubMed, Embase, and CENTRAL were used in December 2024. Eligible RCTs enrolled with IC (excluding critical limb-threatening ischemia) and reported validated HRQoL outcomes at ≥3 months. Two reviewers independently extracted data and assessed risk of bias using the Cochrane RoB 2.0 tool. Random-effects meta-analyses pooled standardized mean differences (SMDs) for HRQoL and mean differences (MDs) for walking distance. **Results**: Five RCTs (n = 728) were included. Compared with optimal medical therapy, both SET and ER improved HRQoL and walking distance. At 12 months, no significant effect was observed between SET and ER (SMD 0.02; 95% CI: −0.18 to 0.22). ER+SET was superior to SET alone (SMD 0.35; 95% CI: 0.12–0.57). Beyond 24 months, improvements were sustained with SET but attenuated with ER, accompanied by higher reintervention rates in ER-containing arms (approximately 20–30% by 2 years). Adverse events were rare (<1%). **Conclusions**: Given moderate-certainty evidence (GRADE), SET should remain the first-line therapy for intermittent claudication because it provides durable improvements in patient-centered outcomes with minimal harm. Endovascular revascularization (ER) can provide faster symptom relief, but its long-term benefits are constrained by restenosis and repeat procedures, particularly in femoropopliteal disease.

## 1. Introduction

Peripheral artery disease (PAD) affects an estimated 236 million people worldwide, reflecting a steady increase from earlier reports, with prevalence increasing with age and major risk factors including smoking, diabetes, hypertension, and dyslipidemia [1,2,3,4]. PAD is a systemic atherosclerotic condition that most commonly affects the lower extremities. Intermittent claudication (IC) is the most common symptomatic presentation and is defined by reproducible exertional leg pain or cramping that resolves with rest. Although not immediately limb-threatening, IC reduces mobility, limits independence, and worsens health-related quality of life (HRQoL) [5]. Patients with IC have a two- to sixfold higher risk of myocardial infarction, stroke, and cardiovascular death than the general population [4,5].

The societal and economic burden of IC is substantial. Patients often reduce physical activity, leading to progressive deconditioning, social isolation, depression, and loss of employment. Healthcare systems incur increased costs due to repeated outpatient visits, diagnostic procedures, and interventions [6]. Accordingly, interventions that improve both symptoms and HRQoL are clinically important. Mechanistically, exercise training can improve claudication-related disability through both central and peripheral adaptations: improved endothelial function and nitric oxide bioavailability, reduced inflammatory signaling, enhanced skeletal muscle oxidative capacity and mitochondrial efficiency, improved microvascular perfusion, and increased tolerance to ischemic discomfort. These effects can translate into sustained functional gains and HRQoL improvements when adherence is maintained. By contrast, revascularization primarily delivers rapid symptom relief by restoring conduit flow, but its long-term value depends on lesion anatomy and patency durability; restenosis and repeat procedures can attenuate early HRQoL gains, particularly in femoropopliteal disease. In practice, SET protocols in RCTs are predominantly supervised walking (treadmill or track) performed to near-maximal claudication pain, while alternative modalities (cycling, resistance training, or mixed programs) exist but are less consistently represented across randomized comparisons in IC [3,7].

Optimal medical therapy (OMT), including antiplatelet agents, statins, antihypertensives, and smoking cessation, forms the cornerstone of management [6]. Two main strategies are available: SET and endovascular revascularization (ER) [1,2].

SET is consistently recommended as first-line therapy in European and U.S. guidelines [1,2]. Programs usually consist of treadmill walking for 30–60 min, for at least three sessions per week, for ≥12 weeks [7]. Benefits stem from improved endothelial function, angiogenesis, enhanced skeletal muscle metabolism, and increased tolerance to ischemic pain [8]. Numerous RCTs have demonstrated SET improves walking distance, functional status, and HRQoL compared with OMT alone. Benefits can be durable when long-term adherence is maintained [9]. Implementation is limited by access to supervised programs, reimbursement constraints, and long-term adherence [10].

ER provides anatomical correction of stenotic or occluded vessels, restoring perfusion and offering faster symptom relief [11]. Techniques include balloon angioplasty, bare-metal and drug-eluting stents, and drug-coated balloons [12,13,14,15]. Outcomes vary by lesion site: iliac interventions are highly durable, whereas femoropopliteal interventions are more prone to restenosis and loss of patency over time [15,16,17]. Complications are uncommon, occurring in 2–5% of cases, with hematoma and distal embolization being the most frequent. Major amputations are rare (<1%) [17,18].

Several RCTs have compared SET, ER, or their combination.

The Claudication: Exercise Versus Endoluminal Revascularization (CLEVER) trial compared SET, iliac stenting, and OMT. At six months, SET improved peak walking time more than stenting, though both improved HRQoL compared to OMT [12]. The Endovascular Revascularization and Supervised Exercise (ERASE) trial randomized patients to ER+SET or SET. ER+SET was superior in HRQoL at 12 months, but at 5 years, outcomes converged due to high reintervention rates in ER groups [14]. The Supervised Exercise Therapy vs. Endovascular Revascularisation for Intermittent Claudication Caused by Iliac Artery Obstruction (SUPER) trial compared SET with iliac stenting, finding a modest but non-significant effect on HRQoL at 12 months [19]. Mazari et al. studied femoropopliteal disease, randomizing patients to SET, ER, or combination therapy. Short-term combined therapy improved outcomes, but long-term follow-up showed no sustained superiority of ER, and higher reintervention rates were observed [17,18]. The Invasive Revascularization or Not in Intermittent Claudication (IRONIC) trial compared invasive and conservative strategies, showing initial QoL gains with invasive therapy, but convergence with SET after 1 year [15,16,17].

Prior systematic reviews concluded that endovascular revascularization (ER) is not superior to SET for improving health-related quality of life (HRQoL) or walking performance, while ER+SET may provide larger short-term gains without sustained benefits beyond 1–2 years [20,21].

The 2024 European Society for Vascular Surgery (ESVS) guidelines [1] and the 2024 ACC/AHA PAD guidelines [2] recommend SET as the initial treatment for intermittent claudication (IC). ER is reserved for patients with lifestyle-limiting symptoms refractory to SET, particularly when lesions are anatomically suitable (e.g., iliac disease). Despite this, in clinical practice, ER is often performed before SET, largely due to limited SET availability and patient preference for immediate symptom relief [1,2].

Given these uncertainties, we conducted a systematic review and meta-analysis of randomized controlled trials comparing SET, ER, and ER+SET. The primary outcome was HRQoL at ≥12 months (and ≥24 months when available), measured using validated instruments. Secondary outcomes included walking performance, reintervention rates, and adverse events.

## 2. Methods

### 2.1. Study Design

We included only randomized controlled trials (RCTs). Non-randomized trials, registries, observational cohorts, case series, and single-arm studies were excluded.

Although this review was not prospectively registered (e.g., PROSPERO), the eligibility criteria, outcomes, and statistical approach were specified before data extraction, and the full database search strategies and analytic decisions are reported transparently to support reproducibility and reduce reporting bias.

We included adult patients (≥18 years) with intermittent claudication (IC) due to peripheral artery disease (PAD). Patients with critical limb-threatening ischemia (CLTI), rest pain, or tissue loss were excluded to maintain homogeneity and to avoid mixing patient populations with different prognoses and treatment goals.

Interventions and comparators: SET structured, supervised programs (typically treadmill or track walking, 30–60 min per session).

Endovascular revascularization (ER): Included balloon angioplasty, bare-metal stenting, drug-eluting stents, or drug-coated balloons.

Combination therapy (ER+SET): Patients treated with ER who were subsequently enrolled in SET programs.

Prespecified comparisons of interest were SET vs. ER, SET vs. ER+SET, and ER vs. ER+SET.

The primary outcome was health-related quality of life (HRQoL). Secondary outcomes included walking performance, reintervention rates, and safety endpoints:

Primary outcome: Health-related quality of life (HRQoL) was measured with validated instruments (e.g., VascuQoL, WIQ, VQ-6, SF-36, EQ-5D).

Secondary outcomes: Functional performance (pain-free and maximum walking distance), rates of reintervention, and safety endpoints (procedural complications, amputations, and all-cause mortality).

Only studies with a minimum follow-up of 3 months and with HRQoL data were included. Outcomes were grouped into short-term (≤12 months), intermediate (12–24 months), and long-term (>24 months).

### 2.2. Search Strategy

A systematic search was performed in PubMed/MEDLINE, Embase, and the Cochrane Central Register of Controlled Trials (CENTRAL) up to 31 December 2024. The search combined controlled vocabulary and free-text terms, without language restrictions, using Boolean operators.

Embase search strategy: (‘intermittent claudication’/exp OR ‘peripheral arterial disease’/exp) AND (‘exercise therapy’/exp OR ‘supervised exercise’ OR ‘structured exercise’) AND (‘endovascular procedures’/exp OR angioplasty OR stent)

CENTRAL search strategy: (“intermittent claudication” OR “peripheral arterial disease”) AND (“exercise” OR “supervised exercise” OR “structured exercise”) AND (“endovascular” OR angioplasty OR stent)

In brief, search terms included the following:

(“Peripheral artery disease” OR “PAD” OR “intermittent claudication”)

AND (“exercise therapy” OR “supervised exercise” OR “walking program”)

AND (“endovascular” OR “angioplasty” OR “stent” OR “revascularization”)

AND (“randomized controlled trial” OR “RCT”).

Additionally, the reference lists of prior systematic reviews [20,21,22,23,24,25] and recent PAD guidelines [1,2] were screened for eligible studies.

### 2.3. Study Selection

**Figure 1:**
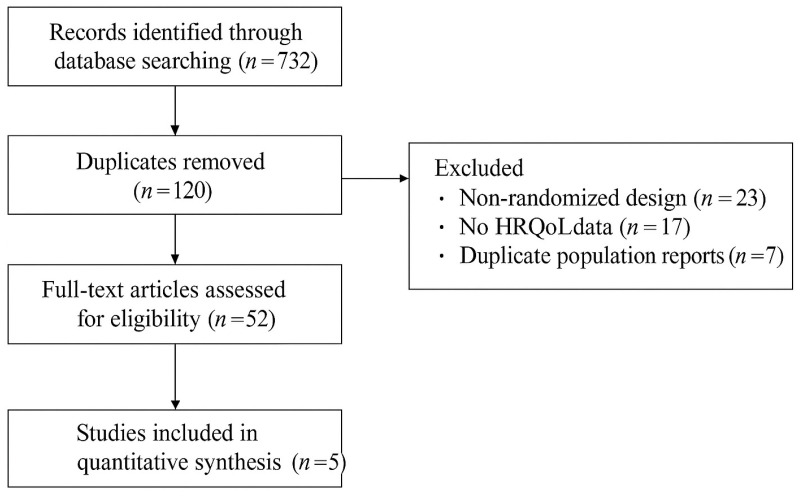
PRISMA flow diagram.

Two reviewers independently screened titles and abstracts, and full-text articles were retrieved for all potentially eligible trials. Disagreements were resolved by discussion and, if necessary, consultation with a third reviewer. The study selection process is shown in the PRISMA 2020 flow diagram (Figure 1) [26,27].

Records identified through database searching (n = 732) were deduplicated (n = 120 removed), leaving 612 records for title/abstract screening. After screening, 52 reports were sought for retrieval and assessed for full text. Forty-seven reports were excluded at full text, primarily due to non-randomized design (n = 23), absence of HRQoL outcomes (n = 17), or duplicate population reporting (n = 7). Five RCTs met all eligibility criteria and were included in the qualitative synthesis and meta-analysis [12,14,15,16,17,18,19].

### 2.4. Data Extraction

Data were extracted independently by two investigators using a standardized extraction form. Extracted variables included the following: trial characteristics (year, country, sample size), patient demographics and baseline ankle–brachial index (ABI), lesion distribution (aortoiliac, femoropopliteal, infrapopliteal), intervention details (type of SET protocol, type of ER device), HRQoL measurement instruments, walking performance outcomes, safety endpoints, follow-up duration, and attrition rates. This meta-analysis was conducted using published aggregate data extracted from trial reports. Individual patient data (IPD) were not available.

Where trials reported medians and interquartile ranges, means and standard deviations were estimated using validated methods. HRQoL outcomes measured using different scales were standardized to standardized mean differences (SMDs, Hedges’ g) to facilitate pooling. Before pooling, all HRQoL instruments were oriented in the same direction, such that higher scores consistently indicated better HRQoL; when necessary, scales were inverted to ensure the interpretability of the SMD. For trials reporting only follow-up values, differences between intervention and comparator arms were calculated; when change scores were available, they were preferentially used.

Missing standard deviations for change scores were imputed using conservative assumptions about within-patient correlation. Sensitivity analyses were performed across a plausible range of correlation values (0.3–0.7) to evaluate robustness.

### 2.5. Risk of Bias Assessment

The risk of bias for each included trial was assessed using the Cochrane Risk of Bias 2 (RoB 2) tool [28]. This approach evaluates five domains: randomization process, deviations from intended interventions, missing outcome data, measurement of outcomes, and selection of the reported result. Two reviewers performed assessments independently, with disagreements resolved by consensus. Sensitivity analyses were performed, excluding studies judged to be at high risk of bias [27].

### 2.6. Data Synthesis and Statistical Analysis

For continuous outcomes (HRQoL, walking performance), we calculated pooled standardized mean differences (SMDs) with 95% confidence intervals (CIs). For dichotomous outcomes (reinterventions, complications), we calculated risk ratios (RRs) with 95% CI:s. All analyses used random-effects models with the Hartung–Knapp–Sidik–Jonkman adjustment, given the small number of trials and expected clinical heterogeneity.

Between-study variance (τ^2^) was estimated using restricted maximum likelihood (REML). We quantified heterogeneity using I^2^, interpreted as ~25% (low), ~50% (moderate), and ~75% (high). Prediction intervals were calculated where possible to provide an estimate of the range of true effects across future settings.

Prespecified subgroup analyses examined lesion location (aortoiliac vs. femoropopliteal vs. infrapopliteal), ER device class (plain balloon/bare-metal stent vs. drug-coated balloon/drug-eluting stent), and exercise modality (supervised vs. home-based/tele-supervised).

Sensitivity analyses included fixed-effect models, exclusion of high-risk-of-bias studies, and leave-one-out analyses. Publication bias was assessed by visual inspection of funnel plots and Egger’s regression test when ≥10 studies were available for a given comparison. When <10 studies were available, publication bias could not be formally assessed and was instead discussed qualitatively.

Analyses were conducted in R (v4.3.1) using metafor (v3.8).

## 3. Results

### 3.1. Study Selection and Characteristics

Five RCTs (CLEVER [12], ERASE [14], IRONIC [15,16], Mazari [17,18], and SUPER) [19] fulfilled all inclusion criteria and were included in the quantitative synthesis.

The included trials were conducted between 2010 and 2022, predominantly in Europe and North America. The mean participant age ranged from 62 to 68 years, with approximately 75% being male. Baseline ankle–brachial index (ABI) ranged from 0.56 to 0.72, consistent with moderate-to-severe PAD. Lesion distribution varied: most trials focused on femoropopliteal disease; one trial enrolled aortoiliac lesions, and one included mixed anatomy. Across trials, pharmacotherapy was a guideline-directed background optimal medical therapy and was not randomized between arms; therefore, comparative drug efficacy was outside the scope of this review, Table 1.

### 3.2. Risk of Bias

Sensitivity analyses using fixed-effect models and alternative correlation assumptions for imputed SDs yielded effect estimates of similar direction and magnitude supporting the robustness of the main findings.

#### 3.2.1. Primary Outcome: Health-Related Quality of Life (HRQoL)

##### SET vs. ER

**Figure 2:**
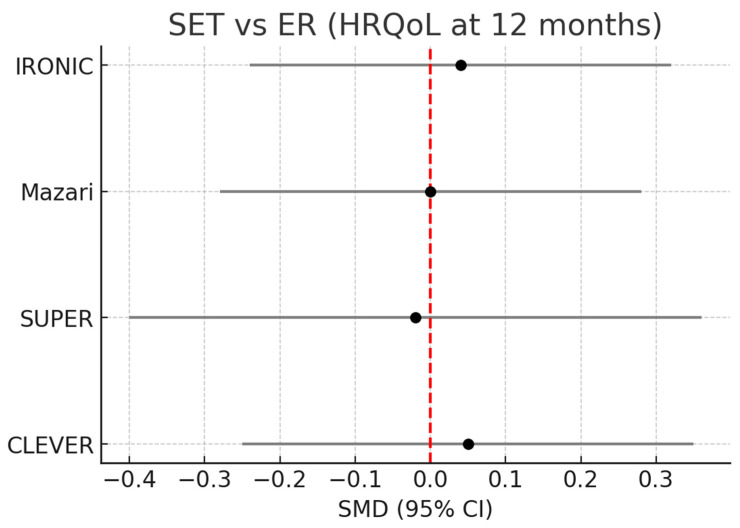
Forest plot: SET vs. ER (HRQoL at 12 months). Forest plot comparing SET versus endovascular revascularization for HRQoL at 12 months (standardized mean difference [SMD], Hedges g; random-effects model with Hartung–Knapp–Sidik–Jonkman adjustment). Positive SMD favors SET after harmonizing HRQoL directionality across instruments.

Four RCTs (CLEVER, SUPER, Mazari, IRONIC) contributed to this comparison and showed no evidence of a clinically meaningful difference between ER and SET at 12 months (pooled SMD ≈ 0; 95% CI crosses 0), Figure 2.

##### ER+SET vs. SET

**Figure 3:**
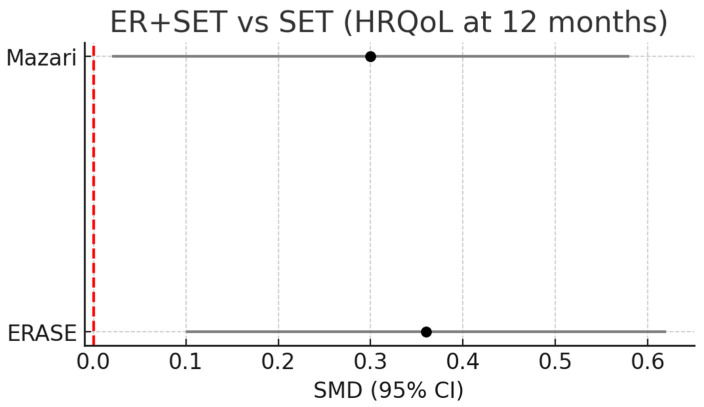
Forest plot: ER+SET vs. SET (HRQoL at 12 months).

Two RCTs (ERASE and Mazari), including approximately 332 patients, showed a statistically significant but clinically modest improvement of ER+SET over SET in HRQoL at 12 months (pooled SMD 0.35; 95% CI: 0.12–0.57), Figure 3.

### 3.3. Long-Term Outcomes

Long-term data (≥24 months) were available from Mazari and IRONIC [15,16,17,18]. These studies showed convergence of HRQoL trajectories between SET and ER-containing strategies, largely due to recurrent interventions and loss of durability in the ER arms. Over five years, SET maintained stable improvements in HRQoL, whereas ER groups demonstrated diminished benefit, with up to approximately 30% of participants in ER-containing arms requiring repeat procedures.

#### 3.3.1. Secondary Outcomes

##### Walking Performance

Two RCTs (ERASE and Mazari), including ~332 patients, demonstrated the significant short-term benefit of ER+SET vs. SET for walking distance (pooled MD 164 m; 95% CI: 98–231; I^2^ = 22%). However, this advantage diminished beyond 24 months due to higher reintervention rates in the ER arms.

Reintervention rates were markedly higher in ER-treated patients. By 24 months, reintervention occurred in 22–30% of ER-treated patients versus near-zero in SET arms, corresponding to a pooled RR of 7.5 (95% CI: 4.1–13.6; I^2^ = 9%).

Procedural complications occurred in 2–5% of ER interventions, most commonly groin hematoma or distal embolization. Major amputations were rare (<1% across all trials). No excess mortality was associated with either strategy. SET carried negligible risk beyond musculoskeletal discomfort.

#### 3.3.2. Subgroup Analyses

Subgroup analyses by lesion location suggested an anatomic hierarchy, but these findings should be considered exploratory due to limited sample sizes:

Aortoiliac disease: ER demonstrated durable improvements, comparable to SET, with low reintervention rates [12,19].

Femoropopliteal disease: ER was associated with higher restenosis and reintervention, explaining the convergence of long-term outcomes [15,16,17,18].

Infrapopliteal disease was not represented in the included intermittent claudication RCTs; therefore, no inference regarding ER versus SET can be made for infrapopliteal anatomy within the present randomized evidence base [11].

Despite angiographic advantages of certain endovascular technologies, these did not consistently translate into HRQoL or functional superiority over SET in the included RCTs [15,18]. Exercise modality (supervised vs. home-based/remote models) was not directly compared within the included RCTs; therefore, statements on home-based or remotely supported programs should be interpreted as implementation considerations rather than trial-derived subgroup effects [3,10,23].

### 3.4. Summary of Findings

Overall, SET and ER both improved HRQoL and walking performance compared with baseline and optimal medical therapy alone. SET and ER were equivalent at 12 months, but SET demonstrated greater durability beyond 24 months due to the absence of restenosis and reinterventions. ER+SET provided superior short-term outcomes compared with SET, but this advantage did not persist in the long term. Safety profiles favored SET, while ER was associated with a procedural risk of 2–5% and a substantial reintervention burden.

Key pooled outcomes and certainty of evidence (assessed by GRADE) are summarized in Table 2 (summary of findings).

## 4. Discussion

This systematic review and meta-analysis of RCTs demonstrates that both SET and endovascular revascularization (ER) improve health-related quality of life (HRQoL) and functional walking capacity in patients with intermittent claudication (IC). However, the durability, safety, and cost-effectiveness of SET establish it as the cornerstone of therapy. ER provides rapid symptomatic relief but is undermined by restenosis and repeat procedures, limiting its long-term value—particularly in femoropopliteal disease. Combination therapy accelerates short-term recovery but offers no sustained superiority over SET alone. Taken together, and recognizing that the certainty of evidence for patient-reported HRQoL and walking outcomes is moderate (GRADE), these findings support a pragmatic treatment pathway aligned with current guidelines: all patients should receive optimal medical therapy, followed by SET as the preferred initial intervention; endovascular revascularization (ER) should be reserved for patients with lifestyle-limiting symptoms that persist despite SET, particularly when lesions are anatomically favorable (e.g., iliac disease). Expanding access to SET programs—including tele-supervised models supported by local evidence and implementation capacity—may help reduce the current evidence–practice gap. Ultimately, these findings support health-system investment in structured exercise therapy to improve durable patient-centered outcomes and promote more sustainable use of healthcare resources, while acknowledging the moderate certainty of the available randomized evidence.

### 4.1. Patient-Centered Outcomes as the Benchmark

Unlike earlier PAD research that often prioritized angiographic patency or treadmill performance, our analysis centers HRQoL as the primary endpoint. This focus aligns with contemporary principles of value-based healthcare, where interventions should be judged not only by technical success but by their ability to improve patients’ daily functioning and quality of life. The consistent finding of SET producing durable improvements in HRQoL highlights the importance of non-invasive strategies that foster physiological adaptation—improved endothelial function, collateral formation, and skeletal muscle efficiency—rather than anatomical correction alone [8,25].

### 4.2. Comparison with Previous Evidence Syntheses

Our findings are consistent with and extend prior systematic reviews. The 2018 Cochrane review concluded that ER offered no superiority over SET in either HRQoL or functional outcomes [20]. Similarly, the 2019 network meta-analysis suggested that ER+SET provided the greatest benefit at 6–12 months, but without long-term advantage over SET alone [21]. By incorporating newer trials, including the SUPER trial of iliac disease and long-term follow-up from ERASE and Mazari [14,21,22,23,25], our review provides an updated synthesis and confirms the durability of SET’s benefits.

Importantly, our subgroup analyses underscore that the long-term equivalence between SET and ER is not simply due to insufficient data but reflects reproducible patterns across lesion types and devices. For aortoiliac disease, ER demonstrates durability and can be justified in selected patients [19,23]. In contrast, femoropopliteal interventions carry a high risk of restenosis and reintervention, eroding their early benefit [15,16,17,25]. Device innovations such as drug-coated balloons and drug-eluting stents reduce angiographic restenosis [14,15,16], but this advantage has not consistently translated into sustained HRQoL improvements, underscoring the limitation of surrogate endpoints.

Safety outcomes favored SET, with no major adverse events attributable to exercise beyond musculoskeletal discomfort. ER strategies carried procedural risks (typically 2–5%) and a substantial reintervention burden, which plausibly explains the convergence of long-term HRQoL trajectories. Although the evidence of cost-effectiveness remains limited, the available data generally support SET as a high-value first-line strategy in systems where structured programs are accessible. Expanding SET capacity (including reimbursed, scalable, and remotely supported models where evidence is available) should therefore be prioritized, while avoiding overstatement that remote delivery is equivalent across all PAD populations [7,29,30]. Aligning reimbursement with evidence would not only improve patient outcomes but also reduce unnecessary procedures, suggesting a greater emphasis on value-based care, consistent with moderate-certainty evidence (GRADE). We did not compare pharmacologic therapies because the review question and eligible trials focused on SET and/or ER strategies on a background of contemporary medical therapy; drug regimens were not randomized consistently across trials, precluding a valid comparative synthesis.

#### Clinical Decision-Making and Treatment Algorithm

Our synthesis supports a pragmatic algorithm:

All patients with IC should receive optimal medical therapy, including antiplatelet agents, statins, and risk factor modification [6].

SET should be offered as the first-line intervention for symptom relief, given its safety, durability, and cost-effectiveness.

ER should be considered for patients who remain severely symptomatic despite SET, particularly those with favorable anatomy such as isolated aortoiliac lesions.

Combination ER+SET may accelerate early gains but does not confer sustained advantage and should be reserved for selected cases.

This algorithm balances patient-centered outcomes with system-level sustainability, ensuring that invasive resources are allocated to those most likely to benefit.

### 4.3. Certainty of Evidence

Using the GRADE framework, the certainty of evidence for HRQoL at 12 months was rated moderate, reflecting the small number of trials and the inherent limitations of unblinded, patient-reported outcomes despite low statistical heterogeneity. Evidence beyond 24 months was downgraded to moderate due to attrition and reduced sample sizes. Walking performance outcomes were rated with moderate certainty due to heterogeneity in measurement protocols. Evidence for reinterventions was high, given consistent and large effect sizes across trials. Adverse event data were judged with moderate certainty, limited by low event counts but consistent directionality.

## 5. Limitations

Several limitations merit consideration. First, the evidence base is limited to five randomized controlled trials (n = 728), which restricts precision, subgroup inference, and assessment of small-study effects. Second, infrapopliteal disease was not represented in the included intermittent claudication RCTs; therefore, these findings should not be extrapolated to infrapopliteal anatomy or to critical limb-threatening ischemia. Third, the protocol was not prospectively registered (e.g., PROSPERO); however, we adhered to PRISMA 2020 guidelines, prespecified all methods, and provided the full search strategy and extraction details. Lack of prospective registration may increase the risk of selective reporting; we mitigated this by prespecifying outcomes and reporting the complete search strategy and analytic framework. Fourth, blinding was not feasible in any trial, which introduced potential expectation bias in patient-reported HRQoL outcomes, while attrition in long-term follow-up may have biased results toward more adherent participants. Fifth, this analysis relied on published aggregate data rather than individual patient data, preventing patient-level analyses. For walking outcomes, arm-level mean±SD values were not consistently available, and published mean differences were used instead. Finally, heterogeneity in exercise testing protocols and HRQoL instruments complicates direct comparisons, although the use of standardized mean differences and random-effects models with Hartung–Knapp–Sidik–Jonkman adjustment mitigates this limitation.

## 6. Future Research

Future trials should address three key gaps:

Standardization of HRQoL instruments: Adoption of disease-specific tools such as VascuQoL or VQ-6 would enable better cross-trial comparability.

Long-term durability: Few RCTs extend beyond two years, and additional follow-up is required to clarify outcomes at five years and beyond.

Implementation science: Pragmatic trials of tele-supervised or community-based SET programs are needed to overcome access barriers and to establish cost-effectiveness in real-world settings.

## 7. Conclusions

In randomized evidence for intermittent claudication, SET provides durable improvements in HRQoL and walking capacity with minimal harm and should remain as a first-line therapy. Endovascular revascularization offers faster symptom relief, but its long-term patient-centered benefit is constrained by restenosis and repeat interventions, particularly in femoropopliteal disease. Combined ER+SET improves short-term outcomes versus SET alone at 12 months, but current randomized data do not demonstrate sustained long-term superiority; future trials should standardize HRQoL reporting, extend follow-up, and test scalable delivery models (including remotely supported SET) alongside cost-effectiveness.

## Figures and Tables

**Table 1:** Characteristics of included RCTs.

Study	Year	Country	N	Baseline ABI	Lesion Location	Interventions
CLEVER	2012	USA	111	0.56–0.72	Aortoiliac	SET vs. ER vs. OMT
ERASE	2015	Netherlands	212	≈0.60	Femoropopliteal	SET vs. ER+SET
IRONIC	2014–2016	Sweden	159	≈0.65	Mixed	SET vs. ER
Mazari	2012–2017	UK	151	≈0.60	Femoropopliteal	SET vs. ER vs. ER+SET
SUPER	2022	Netherlands	95	≈0.68	Iliac	SET vs. ER

**Table 2:** Summary of findings (GRADE).

Outcome	Comparison	Effect Size (95% CI)	Follow-Up	GRADE Certainty
HRQoL	SET vs. ER	SMD 0.02 (−0.18 to 0.22)	12 months	Moderate
HRQoL	ER+SET vs. SET	SMD 0.35 (0.12–0.57)	12 months	Moderate
HRQoL	SET vs. ER	Convergent outcomes	≥24 months	Moderate
Walking distance	SET vs. ER	MD 6 m (−42 to 54)	12 months	Moderate
Walking distance	ER+SET vs. SET	MD 164 m (98–231)	12 months	Moderate
Reintervention	SET vs. ER	RR 7.5 (4.1–13.6)	24 months	Moderate
Adverse events	SET vs. ER	2–5% vs. negligible	Procedural	Moderate

## Data Availability

No new data were created or analyzed in this study.
